# Insights into pubertal development: a narrative review on the role of epigenetics

**DOI:** 10.1007/s40618-024-02513-0

**Published:** 2024-12-20

**Authors:** Anna-Mariia Shulhai, Anna Munerati, Marialaura Menzella, Paola Palanza, Susanna Esposito, Maria Elisabeth Street

**Affiliations:** 1 Pediatric Clinic, Department of Medicine and Surgery, University Hospital of Parma, University of Parma, Parma, 43126 Italy; 2 Department of Pediatrics №2, Ivan Horbachevsky Ternopil National Medical University, Ternopil, Ukraine; 3 Unit of Neuroscience, Department of Medicine and Surgery, University of Parma, Parma, 43125 Italy

**Keywords:** Puberty, Epigenetics, DNA methylation, MiRNA, Histone modification, Chromatin remodeling

## Abstract

**Purpose:**

Puberty is a key phase of growth and development, characterized by psychophysical transformations. It is driven by a combination of genetic, hormonal, and environmental variables. Epigenetic mechanisms, including histone post-translational modifications and chromatin remodeling, microRNAs, and DNA methylation, play important roles in orchestrating the developmental processes. We describe environmental factors that may interact with genetics, and factors influencing puberty onset, focusing in particular on epigenetic mechanisms that can help understand the timing and variations that lead to precocious or delayed puberty.

**Methods:**

We conducted a narrative review of associations between puberty and epigenetic mechanisms through a comprehensive search of PubMed, Scopus, and Web of Science databases.

**Results:**

The chromatin landscape of genes as *KISS1* has revealed dynamic changes in histone modifications as puberty approaches, influencing the stimulation or inhibition of gene expression critical for reproductive maturation. MiRNAs regulate gene expression, whereas DNA methylation affects activation or repression of gene transcription of genes involved in pubertal timing. Moreover, studies in animal models have provided insights into the role of DNA methylation and miRNAs in brain sexual differentiation, highlighting the active involvement of epigenetic mechanisms in shaping sexually dimorphic brain structures.

**Conclusion:**

This review highlights the importance of understanding the complex interplay between epigenetic regulation and pubertal development, which can lead to new therapeutic options and shed light on the fundamental processes driving reproductive maturation.

## Introduction

The intricate process of human growth is regulated by the interplay among genetic factors, endocrine system, environment, and epigenetic control of gene expression. This latter has been more recently recognized. The endocrine system, which releases hormones guiding puberty, undergoes complex regulation through epigenetic mechanisms [1].

Puberty is a transformative stage marking the journey from childhood to adulthood and requires perfect coordination of biological processes depending on gene activation or inactivation, mainly driven by epigenetic regulators [2] that are increasingly gaining attention. Unraveling these mechanisms will help understand the timing of puberty, and its variants leading to precocious or delayed onset of puberty.

Epigenetics has been defined as «the inheritance of variation (-genetics) above and beyond (epi-) changes in the DNA sequence» [3]. So, these processes may modify gene activity without DNA sequence modification. Epigenetic regulation primarily includes genomic imprinting, gene knockdown, and transcriptional regulation of tissue-specific genes during cellular differentiation [2, 4]. These molecular conductors, such as DNA methylation, histone modifications, and non-coding RNA (miRNA), influence gene expression and determine whether certain genes are turned on or off. DNA methylation refers to the addition of methyl groups to DNA molecules, generally resulting in the repression of gene transcription. Histone modifications, alter the structure of proteins around which DNA is wound, influencing how tightly or loosely DNA is packed and, consequently, gene accessibility. Non-coding RNAs, such as microRNAs, act as molecular conductors, fine-tuning gene expression by binding to messenger RNAs and regulating their translation into proteins. Additionally, chromatin remodeling, a dynamic process of altering chromatin structure, plays a role in controlling gene accessibility [4]. Epigenetics is crucial to understanding diseases and could be helpful in future clinical practice. Epigenetic changes can serve as markers and early warning signs, offering insights into the predisposition and development of various processes and diseases [1, 3, 5].

Understanding the epigenetic basis of the onset of puberty provides valuable insights into normal development and unveils potential therapeutic targets for cases of delayed or precocious puberty.This review focuses on the current knowledge related to the interplay between epigenetics and normal puberty and deepens the factors influencing the onset and timing of precocious and delayed puberty.

## Materials and methods

Studies evaluating associations between puberty and epigenetic mechanisms were searched in PubMed, Scopus, and Web of Science databases, from inception to February 2024. The search strategy contained the following terms: «puberty,» «puberty timing,» «precocious puberty,» «delayed puberty,» «epigenetics,» «miRNA,» «DNA methylation,» «histone modifications,» and «chromatin remodeling.» The reference lists of potentially eligible articles were thus screened beforehand.

### Physiology of Pubertal onset

Puberty is a key stage of growth and development, and marks the transition from childhood to adulthood. As previously mentioned, it is driven by a complex interplay of genetic, hormonal, and environmental factors, associated with psycho-physical metamorphoses [2, 6–8]. Studies have shown that in the human body the expression of 927 different genes is related to different ages, and thus stages of development and life. Specifically, a higher expression of genes is observed before the age of 6 years (408 probes, representing 276 genes), with a subsequent reduction as age increases (252 probes, representing 207 genes during puberty). These findings prove a tissue-independent genetic program for human growth and pubertal development [1, 9]. Pubertal onset in girls is characterized by breast development that can begin between the ages of 8 and 13.5 years, pubic hair growth, and accelerated growth velocity. Girls’ puberty ends with menarche, which is the first menstruation followed by attainment of final height due to epiphyseal growth plate closure induced by estrogen [7, 8]. Boys typically experience genital development between the ages of 9 and 14 year, when testicular volume exceeds 4 mL, followed by pubic hair growth and enhanced growth rate. Physiologically, the onset of puberty is caused by the reactivation of signals established throughout fetal life [2, 6]. The onset of puberty is tightly regulated by a network of genes, each playing a specific role in the activation of the hypothalamic-pituitary-gonadal (HPG) axis. The HPG axis is active from birth to 4–6 months in males and up to 2 years in females. This phase is defined «mini-puberty», and is characterized by a loss of negative feedback on the gonadotropin-releasing hormone (GnRH) pulse generator due to the drop in maternal sex hormone levels, with subsequent increase in circulating gonadotropins. In males, increased luteinizing hormone (LH) and follicle-stimulating hormone (FSH) levels lead to further development of the testes and penis, and in females, they are hypothesized to favour breast development, uterine growth and maturation and atresia of ovarian follicles [10, 11]. Following this period, the GnRH pulse generator stops working until reawakening at adolescence [2, 7]. Pubertal activation of GnRH neurosecretion is caused by a decrease in inhibitory transsynaptic inputs and by an increase in transsynaptic and glial excitatory inputs to the GnRH neural network, *KiSS 1* gene, e.g. through kisspeptin and the KiSS1-derived peptide receptor [12]. In response to GnRH, the anterior pituitary presents an increase in the pulsatile release of LH and FSH. These hormones, in turn, stimulate the maturation of reproductive organs and the development of secondary sexual characteristics through the production of testosterone in the testes, and estrogen and progesterone in the ovaries [2, 12, 13].

Precocious puberty represents an early and accelerated onset of sexual development before the age considered normal. This condition is often linked to the premature activation of the HPG axis, resulting in the early release of sex hormones. Genetic factors, such as mutations in genes as Makorin Ring Finger Protein 3 (*MKRN3*) and Delta-like non-canonical Notch ligand 1 (*DLK1*) gene, and central nervous system abnormalities can contribute to precocious puberty [2]. Additionally, environmental influences, including exposure to endocrine-disrupting chemicals, may play a role in disrupting the delicate balance of hormonal regulation [14]. The consequences of precocious puberty extend beyond physical changes, impacting the emotional and psychological well-being of affected individuals [15].

Conversely, delayed puberty is characterized by a slower onset of sexual development, typically after the age considered normal. Delayed puberty may be due to both persistent and functional factors. Persistent factors can be congenital anomalies in the HPG axis or acquired conditions, for example a central nervous system injury causing hypogonadism, hypergonadotropic or hypogonadotropic depending on the site of the damage [7, 16]. Constitutional delay of puberty is typically a simple delay in the activation of the HPG axis for which some genetic causes have been found in recent years. Genetic factors, include Heparan Sulfate 6-O-Sulfotransferase 1 (*HS6ST1*), Immunoglobulin Superfamily Member 10 (*IGSF10*), and Gonadotropin-releasing hormone receptor (*GNRHR*) gene variants [9, 17, 18]. Functional factors such as chronic illnesses, nutritional deficiencies, and environmental stressors can play a role in postponing the activation of the HPG axis, thus, delaying the onset of puberty.

The timing of puberty is a highly individualized process influenced by genetic factors, environmental cues, and epigenetic regulation. Although, the pubertal process is known to have a major genetic component, recent research showed epigenetic regulation of these genes. Furthermore, studies suggest that environmental factors, including nutrition, stress, and exposure to endocrine-disrupting chemicals, can influence the epigenetic regulation of the genes associated with pubertal timing [2, 13, 15].

## Environmental factors and pubertal timing

Different studies have shown that family history and social level, child adoption, stress, environmental factors, and COVID-19 have been linked to pubertal timing variations in offspring [15]. The genetic background accounts for approximately 50–80% of pubertal onset and development heterogeneity. Prenatal disorders, including intrauterine growth restriction (IUGR) and being born small gestational age (SGA), can have an impact on pubertal development. Childhood obesity may favor an early onset of puberty. Stressful conditions as the pandemic, can influence the rise in the incidence of precocious puberty and a faster progression rate of puberty compared to pre-pandemic years [15, 19, 20].

Exposure to endocrine-disrupting chemicals (EDCs) can also affect the delicate process of puberty, as they mimic or interfere with the normal functioning of hormones, but when exposure occurs during pregnancy and the early phases of postnatal life, they determine mainly epigenetic changes [21, 22] that modify gene expression in the later phases of life. The presence of EDCs is pervasive in everyday life, and they are derived from plastics, pesticides, personal care items, electronic devices, fabrics, flooring, utensils, etc. EDCs can subtly influence the timing and progression of puberty [22–24]. Transgenerational studies reveal persistent effects of EDCs on altered pubertal timing and hypothalamic gene network functioning, being transmitted through the germ line [23, 24]. Some EDCs can activate the HPG axis and trigger the premature release of sex hormones [22]. Consequently, there is substantial concern regarding the potential influence of some EDCs, such as phthalates and Bisphenol A (BPA), on precocious puberty due to their pervasive usage, rendering widespread exposure of individuals to these compounds highly probable [25, 26]. Some studies have reported associations between phthalate exposure and early onset of puberty. These studies suggested that phthalates with weak estrogen activity may disrupt biological systems, in particular, if acting at critical periods of development [27]. However, not all studies agree on the impact of endocrine disruptors on precocious puberty. These inconsistent findings on phthalates could be owing to different reasons, such as different timing of the exposure to phthalates. Moreover, many factors, such as age and body mass index (BMI), can influence phthalate and BPA levels. Urinary concentrations of phthalate metabolites have been observed to decline with advancing age [28, 29]. Furthermore, BMI and waist circumference have been shown to impact urinary phthalate levels, which exhibit considerable variability in children across different age groups and developmental stages, partly correlating with the timing of adiposity rebound [25]. Di-2-ethylhexyl phthalate urine metabolites are also associated with puberty onset, obesity, and insulin resistance, showing changes linked to age, height, weight, and fat distribution [30].

Childhood obesity is another factor that influences pubertal timing [31, 32]. Essential obesity is the consequence of environmental, social, genetic and behavioral determinants, which as a result cause an imbalance in energy homeostasis, with higher intake of calories compared to energy waste, resulting in the accumulation of adipose tissue and excess body weight. A passive lifestyle, inadequate sleep and sleep deprivation, and consumption of high-calorie, low-nutrient diets are common behavioral contributors to obesity. A meta-analysis has highlighted obesity in girls as a predisposing factor for an early onset of puberty [31]. Many studies have demonstrated a positive association between BMI and early puberty in girls and boys [31–33]. However, some studies have shown that obesity, rather than just being overweight, is linked to delayed puberty in boys. It is hypothesized that as aromatase activity from adipose tissue is increased, estrogen production is increased, and would determine delayed puberty. Other mechanisms, however, have also been shown and are dependent on the increased serum leptin concentrations that have effects both at the hypothalamic and gonadal levels [31, 32, 34]. Adiponectin, an adipokine secreted by mature adipocytes, has been shown to inhibit kisspeptin gene transcription and gonadotropin-releasing hormone (GnRH) secretion by hypothalamic neurons, thus exerting an inhibitory effect on the onset of puberty [35].

In addition, several studies have indicated a positive correlation between pubertal development and psychological well-being among children and adolescents. Early research has shown that psychological stress itself, caused by insecure parental relationships or parental conflict, can affect the timing of puberty. A recent study found that anxiety and other internalizing symptoms in pre-pubertal girls correlate with an early onset of puberty, regardless of maternal education, anxiety, BMI, or ethnicity [31, 36]. Psychological stress in also a feature in adopted children with premature maturation [15].

During the COVID-19 pandemic, a significant rise in the cases of central precocious puberty (CPP) was observed and this phenomenon was widespread, with an increased worldwide incidence [15, 19, 20]. A series of hypotheses were put forward about the potential factors contributing to accelerated pubertal and CPP development, including both direct and indirect impacts. Direct mechanisms of SARS-CoV-2 on the central nervous system would involve the ACE-2 receptors, hematogenous, olfactory routes, and cytokine storm syndrome. Indirect mechanisms would include increased food intake, decreased physical activity, higher BMI, increased use of electronic devices, changes in sleep patterns and melatonin secretion, increased psychological and emotional stress, and potential heightened exposure to certain endocrine-disrupting compounds due to increased indoor isolation [15, 37]. Most studies confirmed that significant lifestyle alterations, such as those imposed by the COVID-19 lockdown, along with the consequent stress, were probably impacting the regulation of pubertal timing. The noteworthy increase in cases of central precocious puberty, observed during the initial wave of the 2020 pandemic appeared to have been reduced in 2021 and 2022, coinciding with the gradual resumption of regular activities [19, 20, 38]. The strong influence of environmental factors during this period of time comes also from the observation that precocious puberty occurred also in girls whose mothers had normal-late menarche [38].

The potential impact of EDCs on delayed puberty is complex [39, 40]. Certain EDCs, such as pesticides, bisphenols, dioxins, insecticides, have been implicated in hormonal disruptions that delay the activation of the hypothalamic-pituitary-gonadal (HPG) axis. For instance, a study conducted in China revealed a delay in menarche onset among girls with detectable levels of bisphenol A compared to those with undetectable levels. However, the majority of studies have primarily linked exposure to bisphenols with CPP. While there is evidence suggesting delayed pubertal onset and menarche in adolescents with elevated levels of urinary mono-3-carboxypropyl phthalate (MCPP), as well as reduced testicular volume in children exposed to high levels of dioxins [39, 40], but these findings remain controversial.

Environmental factors, such as nutrition, stress, and exposure to endocrine-disrupting chemicals, can influence DNA methylation patterns, miRNA expression, and activity of histone acetyltransferases during puberty. These external factors may interact with genetic predispositions, leading to variations in the epigenetic regulation of puberty-related genes [41–43], as described in the following paragraphs.

## Epigenetics of puberty

### DNAmethylation

DNA methylation can influence the activation or repression of gene transcription involved in puberty-related pathways, shaping the timing and progression of this developmental milestone, gonadal development, and hormone regulation [2, 44–46]. DNA methylation involves the transfer of a methyl group onto the C5 position of cytosine to form 5-methylcytosine. Most of the methylation happens on cytosines that come before a guanine nucleotide or CpG sites. DNA methyltransferases (DNMT) and demethylases (TET) catalyze DNA methylation and demethylation, support genomic integrity, and play a role in the silencing or activation of retroelements [47]. DNA methylation patterns may contribute to the regulation of genes that influence the timing of puberty. Methylation in the CpG islands of the *KISS1* gene promoter, along with the addition of the repressive histone mark H3K27me3 by Polycomb group (PcG) enzymes (EED and CBX7), contribute to *KISS1* repression [44]. TET2 is implicated in the transcription of GnRH, maintaining reproductive function [47].

Kisspeptin, encoded by the *KISS1* gene, plays a key role in the HPG axis, which triggers puberty by releasing GnRH. Methylation in the CpG islands of the KISS1 promoter region inhibits gene expression. As a result, a modification in this pattern, such as demethylation of the CpG islands in the promoter, can be linked to GnRH release and, thus, pubertal onset [13, 44]. Demethylation-mediated synthesis of Zinc finger protein 57 (ZFP57), which regulates genomic imprinting, was found to play a role in several studies. The hypomethylation of ZFP57 in pubertal girls aligns with increased KISS1 and GnRH levels [48]. Additionally, duplication of methyl-CpG-binding protein 2 leads to MECP2 duplication syndrome (MDS), which is also associated with CPP, especially in males [49].

Epigenetic modifications in genes, as *DLK1* and *MKRN3*, associated with the timing of sexual maturation can lead to variations in pubertal onset, affecting whether it occurs earlier or later than average. DNA methyltransferase inhibitors (DNMTi) have been demonstrated to prevent pubertal onset [50, 51]. It has been demonstrated recently that *MKRN3* knockout mice present CPP. The *MKRN3* gene regulates the switch to puberty onset by ubiquitinating MBD3, disrupting its binding with the GNRH1 promoter, which recruits TET2. These findings support a role for TET2 as a direct controller of GnRH1 [52, 53]. DLK1 hypomethylation may be considered in patients with sporadic CPP, as in Temple syndrome [54, 55] where CPP has been ascribed to different methylation and expression of the maternal and paternal *DLK1-DIO3* gene cluster, which is controlled by the imprinting control region IG-DMR [56].

Table 1 summarizes the known methylation patterns on genes relevant to pubertal development at the onset of puberty and the enzymes that are known to mediate these processes.

**Table 1:** DNA methylation effects and the enzymes involved in the expression of key genes for pubertal development

Process	Enzyme	Target	Effect	Reference
Demethylation	TET	GnRH	Activation of GnRH	[47]
Demethylation	TET2	MKRN3	Activation of GNRH1 promoter	[52]
Methylation	DNMTs	GnRH	Repression of GnRH	[44, 50]
Methylation	DNMTs	KISS1 gene promoter	Repression of KISS1	[44, 50]
Methylation	DNMTs	EED and CBX7	Activation of KISS1	[50]

DNMTs—DNA methyltransferases enzymes; TET—ten-eleven translocation enzyme; TET2—ten-eleven translocase-2; GnRH—Gonadotropin-releasing hormone; MKRN3—Makorin Ring Finger Protein 3; EED—enhanced enzyme diffusion; CBX7—Chromobox protein homolog 7

Currently, published research discovered a widespread pattern of DNA hypermethylation in girls with both normal and abnormal puberty, demonstrating that the pubertal process in humans is linked to particular changes in epigenetically mediated regulatory regulation [57]. Genome-wide changes in leukocyte DNA methylation in healthy girls and boys during pubertal development, including several differentially methylated genomic regions linked with normal puberty. The most important region for both sexes was found in the Thyroid Hormone Receptor Interactor 6 (TRIP6) promoter region located on 7q22. As a result, the methylation of the TRIP6 promoter may be related to pubertal development. However, there is a lack of knowledge regarding the functional role of human TRIP-6 [58].

A large-scale cohort study of Finish twins identified DNA methylation at 58 CpG sites, affecting both sex-specifically and cross-sex, both positively and adversely, to affect pubertal development progression, and age at the onset of puberty. Twin modeling showed that these CpG sites were highly heritable, driven mostly by genetic and environmental factors. The observed sex differences in methylation highlight the need for sex-stratified models in puberty-related epigenetic investigations, implying potential mechanistic functions that require further investigation [46].

DNA methylation differences between men and women can also impact the sexually dimorphic aspects of puberty [59]. DNA methylation changes contribute to the development of secondary sexual characteristics. Researchers have found that 273 CpGs in girls and 67 CpGs dinucleotides in boys are associated with puberty and pubertal markers, such as breast development in females, growth spurt, and deepening of the voice with facial hair growth in males. Most of the found CpGs were related to the age at menarche in girls, and with facial hair growth in males [60].

Sex hormone genes, such as those of estrogens and testosterone, may show changes in methylation patterns, impacting their expression levels [61, 62]. Around 120 chromosomal sites in females exhibit distinct methylation patterns before and during puberty [48]. More than 40 differentially methylated areas have been found to be connected with sex hormones and SHBG levels in males [62]. Furthermore, DNA methylation and demethylation affect the amount of estrogen receptor alpha (ERα) cells in the arcuate nucleus (ARC), and methylation is associated with ESR1 activation in this region. In female mice, inhibiting DNA methylation decreases ERɑ, while inhibiting demethylation enhances ERɑ expression in male mice. The researchers hypothesized hence that testosterone could program the amount of ERα on cells via epigenetic pathways [61].

Beyond the physiological aspects, DNA methylation is implicated in the epigenetic regulation of genes involved in brain development and function during puberty [63, 64]. This includes genes related to emotional regulation, cognitive abilities, and social behavior, influencing adolescent behavioral changes. As we described, this hypothesis is regulated through ERα, as well as through estrogen receptor beta (ERβ) and progesterone receptors [63]. ERα methylation in the brain increases during early development, possibly initiated by DNMT3a and perpetuated by DNMT1. DNMT3Aa levels peak in mice pups at day 10 and then fall, while DNMT1 levels rise and persist into adulthood. Similar patterns are observed for ERβ. Reduced circulating hormones in aging have been linked with increased methylation and decreased expression of ERβ across the brain [63]. These changes are important to shape sex differences in the brain. A study on female mice using testosterone and DNMT inhibitors during early postnatal stages found that DNA methylation alters the number of calbindin cells in the preoptic area (POA) and reduces the number of ERα cells in the ventromedial nucleus of the hypothalamus, leading to the masculinization of the brain in males [65]. While feminization of the brain has previously been hypothesized to be a passive process, recent studies indicate that feminization in the rat brain actively involves the repression of masculinizing genes via DNA methylation, opening up new paths for epigenetics research [65, 66].

## miRNAs

**Fig. 1:**
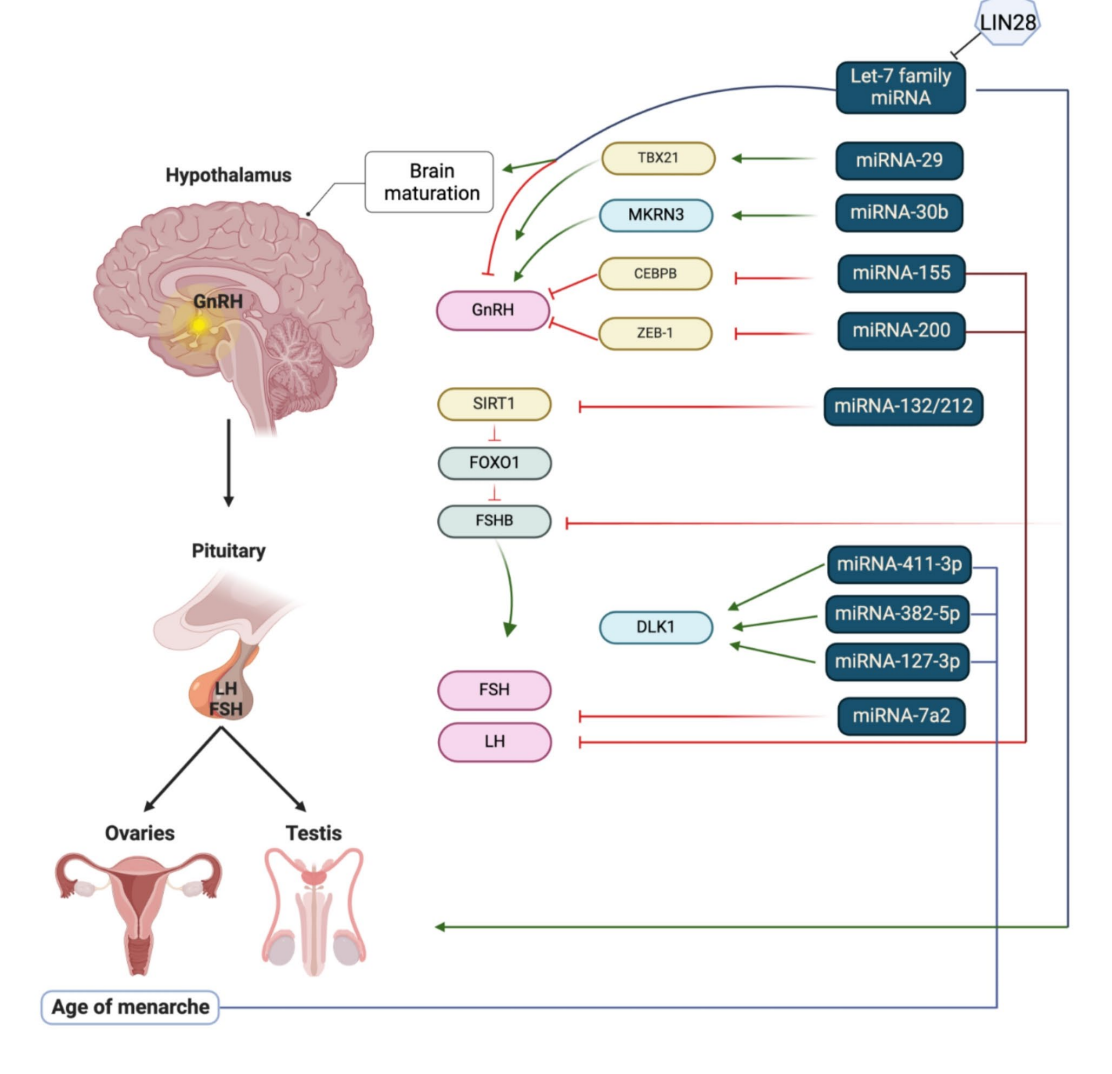
miRNAs known to play key functions in puberty. GnRH - gonadotropin-releasing hormone; LH - luteinizing hormone; FSH - follicle-stimulating hormone; FSHB - follicle-stimulating hormone-β subunit; SIRT1 - sirtuin 1; FOXO1 - Forkhead box O1; TBX21 - T-Box Transcription Factor 21; MKRN3 - Makorin Ring Finger Protein 3; CEBPB - CCAAT/enhancer binding protein beta; ZEB-1 - Zinc finger E-box binding homeobox 1; DLK1 - Delta like non-canonical Notch ligand 1. Red lines show inhibition, green lines increased gene transcription. Created with BioRender.com.

MicroRNAs (miRNAs) are short, non-coding RNA molecules, approximately 24 nucleotides long, that play an important role in post-transcriptional gene regulation. Each miRNAs can regulate hundreds of target mRNAs, and overall, they regulate the expression of at least 30% of the mammalian genome. The genes encoding for miRNAs are typically found in both intergenic and intragenic areas within the human genome. Approximately 25% are encoded in polycistronic transcripts exhibiting a coordinated regulation as they are part of the same regulatory network [1, 4]. They are known to be involved in various biological processes, including development and hormonal regulation. MiRNAs contribute to regulate hormones involved in puberty, such as GnRH, LH, and FSH, as they target their genes within the hypothalamus and pituitary gland, regulating the synthesis and release of these hormones and, subsequently, the onset and progression of puberty. Current knowledge on specific miRNAs and the mode of action are summarized in Fig. 1, and detailed below.

Specifically, miRNA-200 and miRNA-155 regulate GnRH expression and control the expression of GnRH key transcriptional repressors Zinc finger E-box binding homeobox 1 (ZEB-1) and CCAAT/enhancer binding protein beta (CEBPB), a nitric oxide-mediated repressor that acts directly and through ZEB-1. This regulation results in increased transcriptional activation of GnRH and in a decrease in ZEB-1 and CEBPB levels. These changes contribute also to the upregulation of GnRH synthesis in the GnRH neurons. Mice models have shown that impaired miRNA synthesis is associated with decreased plasma LH and FSH levels, i.e., hypogonadotropic hypogonadism, and infertility due to a loss in GnRH expression [67]. Studies have proved that miR-132/212 is important for the up-regulation of FSHβ subunit (FSHβ) synthesis and secretion induced by GnRH, as inhibition of miR-132/212 reduces the up-regulation of FSH secretion by GnRH. MiR-132/212 also targets sirtuin 1 (SIRT1) mRNA, reducing SIRT1 deacetylase levels with subsequent acetylation of FOXO1, which in turn contributes to the stimulation of FSHβ expression [68]. Additionally, miRNA-7a2 is important for normal mouse pituitary development and HPG function, and its deletion in mice leads to hypogonadotropic infertility [18, 69].

MiRNA-30b contributes to the regulation of MKRN3, indicating a unique hypothalamic route impacting pubertal onset [51, 70]. A rodent study found that the expression of hypothalamic miRNA-30b increased during postnatal maturation, when MKRN3 decreased. In vitro analyses showed that miRNA-30b had a strong repressive effect on MKRN3 3′UTR, reducing MKRN3 expression in Kiss1 neurons [51, 70, 71]. A human study of boys with constitutional delay of growth and puberty showed increased miRNA-30b during puberty and its relation with the HPG axis [72, 73].

Changes in Let-7 family miRNA expression in the hypothalamus and the menarche-modulating gene LIN28B, which suppresses the synthesis of Let-7 miRNAs, have been observed in independent genome-wide association studies before puberty [70, 74]. Additionally, the expression of miR-411-3p, miR-382-5p, and miR-127-3p have been linked to age of menarche variability [44, 75]. The increased expression of miRNAs in GnRH neurons during the infantile period contributes to puberty progression [2, 70]. Other studies have shown that the overexpression of LIN28 leads to delayed puberty [74], but the repressive mechanisms in CPP remain ambiguous.

The adolescent brain undergoes profound changes during puberty, and miRNAs play a crucial role in sculpting these neurological transformations. They are involved in synaptic plasticity, emotional regulation, and the maturation of neural circuits, under the regulatory miRNAs influence, contributing to the cognitive and behavioral changes observed during adolescence, mainly through Lin28/let-7 system in the hypothalamus [76, 77]. Moreover, age-related miRNA 3’ shortening was found to be a feature of postnatal brain maturation [78], but to understand the consequences of this requires further studies.

Finally, there is a crosstalk between miRNAs and other epigenetic mechanisms, such as DNA methylation and histone modifications, during puberty. MiRNAs can target components of the epigenetic machinery, influencing the overall epigenetic landscape and gene expression patterns associated with pubertal development. The miRNAs from the miR-29 family mediate TBX21 transcription factor [79] and target TET enzymes that initiate the removal of methylation marks of which we do not yet know the consequences. In somatic cell reprogramming, miR-302 facilitates global DNA demethylation by suppressing various epigenetic regulators, such as MECP2, and DNMT1 through AOF2. Additionally, miR-148a, miR-29, and miR-152 were identified as regulators of DNA methyltransferases, and miRNA-128 expression reduces H3K27me3 mark [80], which is important for pubertal timing.

### Histone modifications and chromatin remodeling

Histone modifications and chromatin remodeling are important epigenetic mechanisms that significantly regulate gene expression during puberty [81]. Histones are proteins around which DNA is wound to form nucleosomes. Histone modifications, such as methylation, acetylation, phosphorylation, ubiquitination, and sumoylation, can alter chromatin’s three-dimensional structure shape [4]. Acetylation of histones is related to transcriptional activation, while deacetylation is associated with transcriptional repression [4]. As puberty develops, specific histone modifications participate in the opening and closing of chromatin, facilitating the activation or repression of genes crucial for the progression of adolescence. This hormonal regulation influences the accessibility of genes involved in pubertal development, such as those related to the production of sex hormones and the maturation of reproductive organs [82].

Chromatin consists of histones and DNA. Chromatin remodeling involves the repositioning, removing, or restructuring nucleosomes, altering the accessibility of DNA to transcriptional machinery [4]. ATP-dependent chromatin remodeling complexes, such as SWI/SNF and ISWI, play a role in this dynamic process. Chromatin remodeling facilitates the unwinding of DNA in these gene regions, making them accessible for transcription and contributing to puberty-related processes [83, 84]. Gonadotropins and sex steroids interact with chromatin remodeling complexes, promoting the activation or repression of target genes essential for the progression of puberty [84].

Puberty is regulated by a complex interplay of epigenetic mechanisms that govern the expression of critical genes in the hypothalamus. Chromatin-modifying complexes with antagonistic activity, such as the Polycomb group (PcG) and the Trithorax group (TrxG), play an important role in this regulation [85, 86]. PcG proteins silence genes and induce repressive chromatin structure by depositing repressive histone marks such as H3K27me3, whereas TrxG proteins reverse this repression by adding activating marks such as H3K4me3 [44, 85, 87]. PcG proteins may suppress the expression of genes implicated in puberty activation, such as *KISS1* and glutamate receptor genes. However, as puberty approaches, PcG activity decreases, resulting in chromatin remodeling characterized by increasing levels of activating histone modifications such as H3K4me3, H3K9/16ac and H3K9/14ac at the *KISS1* promoter, which facilitates gene expression and puberty. Specifically, the enzyme KDM6B has been identified as a key member in this network, erasing PcG-dependent histone modifications and activating puberty-related genes [41, 44, 88]. TrxG members MLL1 and MLL3, which promote gene expression, provide an additional layer of regulation. During puberty, their abundance at the KISS1 promoter contributes to the shift in chromatin structure toward activation. MLL1 knockdown in female rats delays puberty, underlining its significance [44]. During prepuberty, PcG proteins, such as EED and CBX7, inhibit KISS1 expression by generating repressive histone modifications, such as H3K27me3, near its promoter region [41, 44, 88]. However, with the onset of puberty, DNA methylation increases at the promoters of *PcG* genes, increasing transcription-activating histone marks (H3K4me3, H3K9ac, and H3K14ac), with subsequent decreased expression and activation of *KISS1*. On the day of the first surge of gonadotropins, the H3K27me3 histone begins to decrease significantly, and the amount of activating marks such as H3K4me3, which facilitates gene expression, starts rise [41, 88–90].

SIRT1, an enzyme interacting with PcG proteins, reinforces repression by removing histone acetylation. As puberty approaches, SIRT1 dissociates from the *KISS1* promoter, causing a shift in chromatin structure towards an active state. This transition is marked by higher levels of activating histone marks (H3K9/16ac) and lower levels of repressive marks, which allows for increased Kiss1 expression and the onset of puberty [71, 90]. Additionally, SIRT1 interacts with PcGs to stimulate histone methylation at lysine 9 (H3K9me3) and lysine 27 (H3K27me3), further contributing to gene silencing [84].

A lysine-specific histone demethylase 1 A (LSD1) removes methyl groups from di- and mono-methylated H3K4 in chromatin remodeling [91]. Mice model studies have shown that female mice with heterozygous LSD1 deletion exhibit early vaginal opening and first ovulation, which occurs 3–4 days earlier than the wild-type counterparts. This early beginning is marked by elevated plasma gonadotropin levels and enhanced hypothalamus expression of the puberty-related gene Tac2 [92]. It is hypothesized that lower levels of LSD1 lead to higher *KISS1* levels and precocious puberty; however, further investigations are needed.

Furthermore, histone modifications contribute to the development of gender-specific characteristics. For instance, lysine demethylase 5 A (KDM5A) plays a role in the transformation of gonocytes to spermatogonial stem cells by regulating the transcription of specific genes via H3K4me3 [93]. Estrogen changes *KISS1* promoter histone acetylation, and may contribute to the positive feedback involved in the preovulatory surge of LH in females. This dynamic chromatin landscape, characterized by both repressive and activating epigenetic factors, enables swift modulation and control of gene expression [24, 82].

A recent investigation of epigenetic marker levels in pre-pubertal and post-pubertal female mouse oocytes evidenced considerable changes in the chromatin structure and epigenetic landscape [89]. These changes are characterized by changes in histone features, including increased histone methylation and reorganization of chromatin architecture. H3K4me3 staining was less in pre-pubertal oocytes than in post-pubertal oocytes, but H3K27ac was significantly higher. While pre-pubertal oocytes have higher levels of histone acetylation and lower histone methylation than post-pubertal oocytes, exposure to FSH during this transition period led to chromatin remodeling, elevating methylation levels and changes in meiotic function. However, despite changes in chromatin structure, meiosis in pre-pubertal oocytes remains incomplete, possibly due to the saturated histone acetylation pattern induced by FSH [89].

Interestingly, in the latest study of boys with hypogonadotropic hypogonadism and high infertility risk, impaired chromatin remodeling was found. Researchers have shown that this was due to decreased levels of histone deacetylase and increased expression of methyltransferase and Histone deacetylase 8 (HDAC8) decrotonylase [93].

Likewise, histone post-translational modifications contribute to masculinize the preoptic area, with histone deacetylase (HDAC) activity being inhibited during neonatal life reducing male behavior. Specifically, HDAC2 and HDAC4, potentially in conjunction with ERα and aromatase gene promoters, are essential for male-typical behaviors [84]. However, the role of other histone post-translational modifications in brain sexual differentiation requires further exploration.

**Fig. 2:**
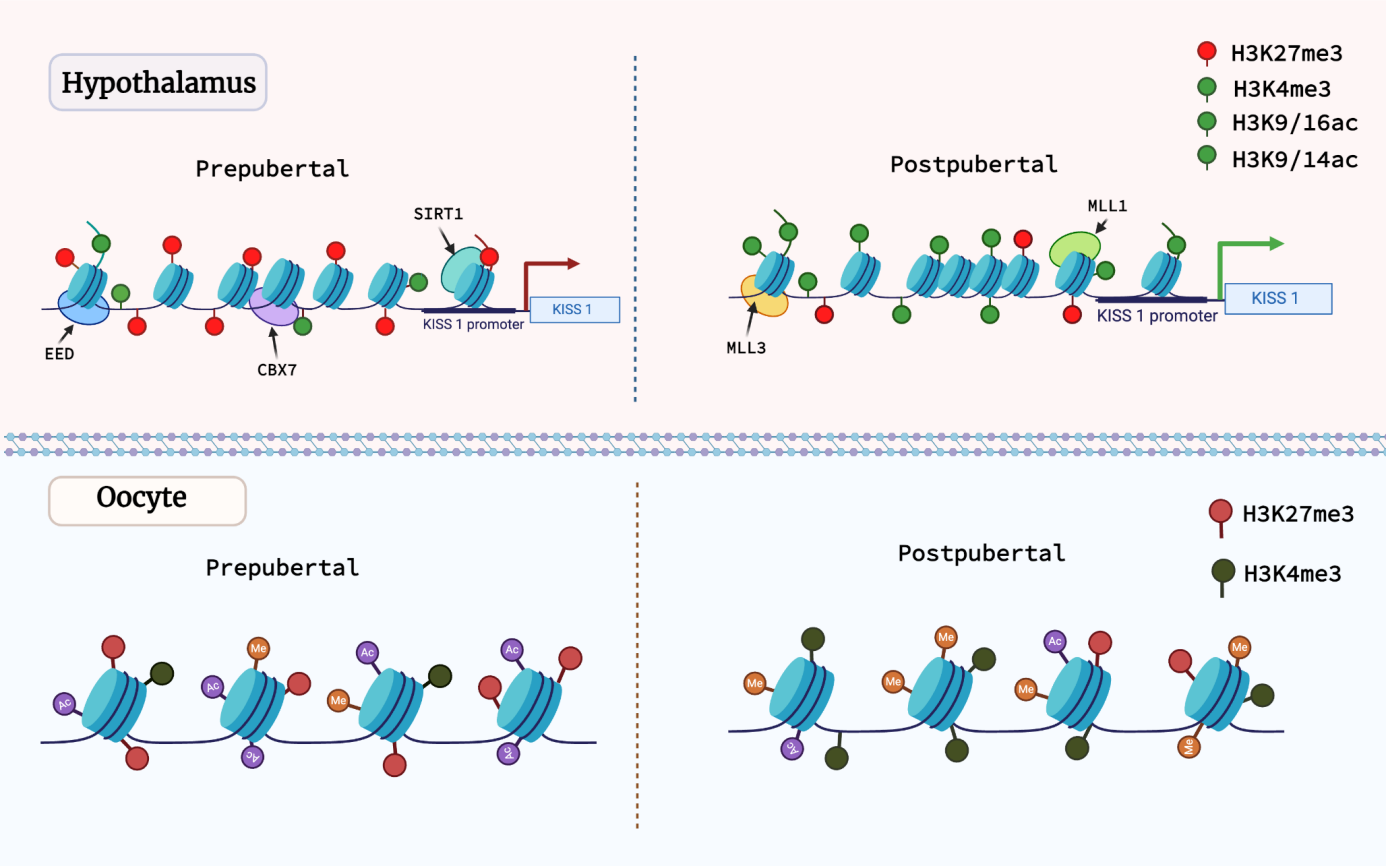
Histone Modifications and chromatin remodeling in the hypothalamus and oocytes during the prepubertal and pubertal periods of life in female mouse models. In the prepubertal period, there is a prevalence of epigenetic repressive histone marks such as H3K27me3 and fewer activating marks such as H3K4me3, H3K9/16ac and H3K9/14ac. With the progression of puberty, there is a decrease in Polycomb group activity, resulting in chromatin remodeling characterized by increasing levels of activating histone modifications. The green circles represent activating histone modification marks, and the red circles repressive histone modification marks. EED - enhanced enzyme diffusion; CBX7 - Chromobox protein homolog 7; SIRT1 - Sirtuin 1; MLL3 - Mixed-Lineage Leukemia 3; MLL1 - Mixed-Lineage Leukemia 1; Me - methylation; Ac - acetylation; H3K27me - Histone 3 Monomethylated at Lysine 27; H3K4me3 - Histone 3 Trimethylated at Lysine 4; H3K9/14ac - Histone 3 acetylated at lysines 9 and 14; H3K9/16ac - Histone 3 acetylated at lysines 9 and 16. Created with BioRender.com.

The main changes in histones in females, both in the hypothalamus and in the oocytes between prepuberty and puberty are summarized in Fig. 2.

## Future directions

The knowledge of epigenetic mechanisms first adds new important information on the mechanisms regulating the onset, progression, and duration of puberty. Second, it leads to the possibility of putting forward a hypothesis of new targeted therapies that can modify epigenetics and open new avenues to regulate/modify pubertal development. Specific epigenetic markers or miRNA expression patterns could potentially be used to address clinical questions related to the diagnosis and treatment of pubertal disorders or imbalances.

Developing therapies that either mimic or inhibit specific miRNAs may become relevant in the future for addressing disorders associated with abnormal miRNA expression, as creating miRNA delivery systems or drugs in combination with miRNAs as has been done in novel cancer therapy [94]. Modulating DNA methylation patterns could be a potential strategy to influence the expression of genes involved in pubertal development. For instance, the first clinical trials using combination treatments with DNMT inhibitors and chemotherapy have given promising results compared to exclusive drug therapy [95]. Furthermore, in cancer, epigenetic regulators have been shown to improve the durability of cancer immunotherapy [96]. Controlling histone modifications could impact the accessibility of genes involved in puberty. Targeting histone acetyltransferases (HATs) and histone deacetylases (HDACs) could offer a way to modify the epigenetic landscape and regulate the expression of genes associated with pubertal development. Integrating data from various omics technologies, including genomics, epigenomics, transcriptomics, and metabolomics, would provide a further comprehensive understanding of the molecular landscape of puberty.

Identifying ways to mitigate negative environmental influences and promote healthy pubertal development may have long-term implications for overall health.

## Conclusions

**Fig. 3:**
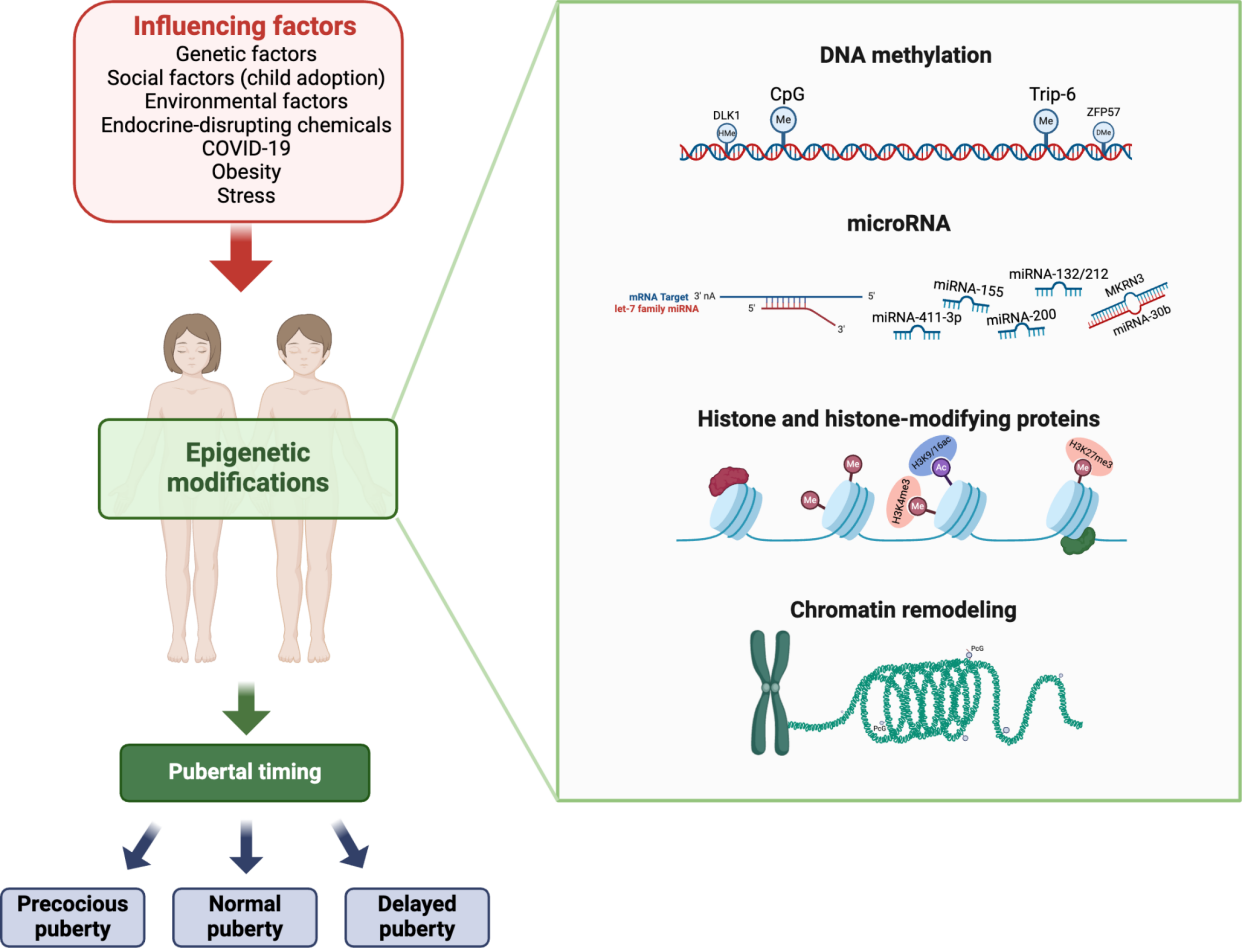
Epigenetics of puberty. The main factors contributing to the epigenetic regulation of puberty are represented on the left side whereas the main mechanisms of epigenetic regulation known to be involved in the onset of puberty are represented on the right side. Created with BioRender.com

The main epigenetic biomarkers known to be involved in puberty are summarized in Fig. 3.

The epigenetics of puberty helps to understand the transition from childhood to adulthood, and deepens the understanding of human pubertal development while providing new insights into the factors influencing health and disease across the lifespan. From DNA methylation to miRNA modulation, these epigenetic mechanisms weave a molecular tapestry that goes beyond the physical changes, influencing cognitive, emotional, and behavioral dimensions. As we unravel the specific epigenetic signatures associated with pubertal timing and development variations, potential new therapeutic targets come to light. Modulating DNA methylation patterns, targeting specific miRNAs, or even manipulating histone modifications could offer precision interventions in pubertal disorders or imbalances in the future to come.

## Data Availability

No code to share.
